# Regulation of the Contribution of Integrin to Cell Attachment on Poly(2-Methoxyethyl Acrylate) (PMEA) Analogous Polymers for Attachment-Based Cell Enrichment

**DOI:** 10.1371/journal.pone.0136066

**Published:** 2015-08-19

**Authors:** Takashi Hoshiba, Eri Nemoto, Kazuhiro Sato, Toshihiko Orui, Takayuki Otaki, Ayano Yoshihiro, Masaru Tanaka

**Affiliations:** 1 Graduate School of Science and Engineering, Yamagata University, Jonan, Yonezawa, Yamagata, Japan; 2 International Center for Materials Nanoarchitectonics, National Institute for Materials Science, Namiki, Tsukuba, Ibaraki, Japan; 3 Department of Biochemical Engineering, Yamagata University, Jonan, Yonezawa, Yamagata, Japan; 4 Institute for Materials Chemistry and Engineering, Kyushu University, Motooka, Nishi-ku, Fukuoka, Japan; Osaka University, JAPAN

## Abstract

Cell enrichment is currently in high demand in medical engineering. We have reported that non-blood cells can attach to a blood-compatible poly(2-methoxyethyl acrylate) (PMEA) substrate through integrin-dependent and integrin-independent mechanisms because the PMEA substrate suppresses protein adsorption. Therefore, we assumed that PMEA analogous polymers can change the contribution of integrin to cell attachment through the regulation of protein adsorption. In the present study, we investigated protein adsorption, cell attachment profiles, and attachment mechanisms on PMEA analogous polymer substrates. Additionally, we demonstrated the possibility of attachment-based cell enrichment on PMEA analogous polymer substrates. HT-1080 and MDA-MB-231 cells started to attach to poly(butyl acrylate) (PBA) and poly(tetrahydrofurfuryl acrylate) (PTHFA), on which proteins could adsorb well, within 1 h. HepG2 cells started to attach after 1 h. HT-1080, MDA-MB-231, and HepG2 cells started to attach within 30 min to PMEA, poly(2-(2-methoxyethoxy) ethyl acrylate-*co*-butyl acrylate) (30:70 mol%, PMe2A) and poly(2-(2-methoxyethoxy) ethoxy ethyl acrylate-*co*-butyl acrylate) (30:70 mol%, PMe3A), which suppress protein adsorption. Moreover, the ratio of attached cells from a cell mixture can be changed on PMEA analogous polymers. These findings suggested that PMEA analogous polymers can be used for attachment-based cell enrichment.

## Introduction

Cell enrichment, a technique used to isolate or concentrate specific cells from a mixture of various types of cells, is currently in high demand because of recent developments in medical engineering. For example, cell enrichment is expected to isolate rare cells, such as endothelial progenitor cells (EPCs) and circulating tumor cells (CTCs), from blood for tumor diagnosis and therapy of vascular failure, respectively [1, 2]. It is also strongly desired for the enrichment of differentiated cells from the differentiation culture of stem cells for regenerative medical applications [3, 4].

Cells are typically enriched using antibody-based methods, such as flow cytometry because of their high specificity [5]. Antibody-based enrichment requires interaction with antibodies to label the cells with high specificity. In spite of the high specificity of antibody-based enrichment, alternative methods are expected to be developed because antibody-based enrichment is not only expensive but also requires a well-trained operator and special apparatus [6]. Additionally, it is desirable to avoid labeling with an antibody for downstream clinical applications [7].

Differential properties of cell attachment to the substrates are often used for cell enrichment [8–11]. For example, hepatocytes specifically attach to galactose-carrying polystyrene, poly(*N*-*p*-vinylbenzyl-4-*O*-β-**D**-galactopyranosyl-**D**-gluconamide) (PVLA) via asialoglycoprotein receptors (ASGP-Rs), which are expressed on the hepatocyte surface, and its specific attachment to a PVLA substrate can be utilized for primary hepatocyte isolation [8]. Mesenchymal stem cells (MSCs) are normally isolated from bone marrow cells according to the different attachment properties of MSCs compared to other cells [9]. Attachment-based enrichment is easy to apply for label-free cell enrichment. Moreover, attachment-based enrichment does not require a well-trained operator or a special apparatus. Therefore, attachment-based enrichment is one of the most useful methods that can be applied in regenerative medicine.

Attachment-based enrichment is usually performed on substrates immobilized with a cell-specific ligand, such as galactose [8]. Although substrates immobilized with a cell-specific ligand are useful for cell enrichment, ligand-dependent attachment-based enrichment is unavailable for the cells whose specific ligands are not identified. Attachment-based enrichment for these cells is performed by methods that depend on the strength of cell attachment to the substrate [9, 10]. However, it is difficult to predict or adjust the strength of attachment to the substrates, thus preventing broad applications of the enrichment for a variety of cells. Cells generally attach to the substrates which do not present any specific ligands via the interaction between integrin and adsorbed proteins (*e*.*g*., fibronectin and vitronectin) [11]. Moreover, cells can attach to the substrates via an integrin-independent mechanism (*i*.*e*., direct interaction with the substrates) in a serum-free medium [12]. The attachment strength between integrin-dependent and integrin-independent mechanisms could be different. Therefore, changes in the contribution of integrin to cell attachment on the substrates lead to variation of attachment strength and allow for broad applications of attachment-based enrichment for a variety of cells.

We have recently reported cell attachment on poly(2-methoxyethyl acrylate) (PMEA) and poly(tetrahydrofurfuryl acrylate) (PTHFA) substrates [13]. Because serum proteins can be well-adsorbed on the PTHFA substrate [13], cells attached to the PTHFA substrate via an integrin-dependent mechanism in serum-containing medium. In contrast, cells attached to the PMEA substrate via both integrin-dependent and integrin-independent mechanisms in serum-containing medium because serum protein adsorption was suppressed and the substrate surface was exposed to cells, allowing direct cell-substrate interaction [13]. Therefore, we hypothesized that PMEA, PTHFA, and their analogous polymers can change the contribution of integrin to cell attachment on the substrates and that these polymers can influence the attachment strength to the substrate through the regulation of protein adsorption and the contribution of integrin to cell attachment.

In this study, we investigated protein adsorption, cell attachment profiles, and attachment mechanisms on PMEA, PTHFA, and their analogous polymer substrates. We demonstrated the feasibility of enriching cells based on attachment to these substrates. To serve as PMEA analogous polymers, we designed polymers that vary in the number of repeated ethylene glycol units of the PMEA side chain [14]. This is expected to alter the hydration condition of these polymers, as well as the integrin-dependent and –independent attachment on these polymer substrates.

## Results

### 2.1. Protein adsorption behavior on PMEA analogous polymer substrates

**Fig 1:**
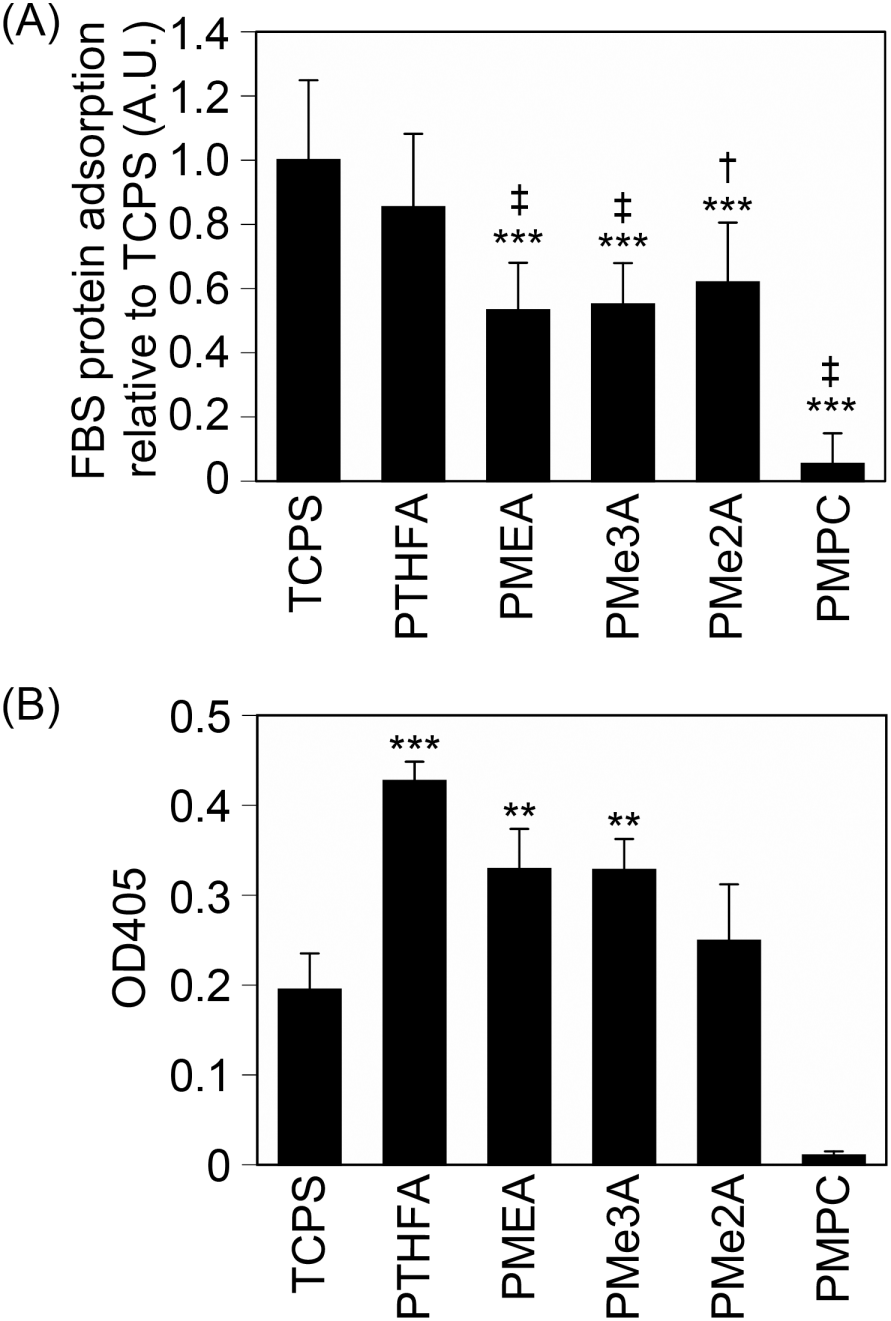
Regulation of protein adsorption by the PMEA-analogous polymer substrates. (A) The amounts of adsorbed protein from the serum-containing medium after 1 h. The data represent the means ± SD. (n = 5). ***: *P* < 0.005 *vs*. TCPS. †: *P* < 0.05 *vs*. PTHFA, ‡: *P* < 0.01 *vs*. PTHFA. (B) Exposed cell attachment sites in FN adsorbed onto the PMEA-analogous polymer substrates. The data represent the means ± SD (n = 4). **: *P* < 0.01, ***: *P* < 0.005 *vs*. TCPS.

Protein adsorption strongly impacts cell attachment to polymer substrates in serum-containing medium. Therefore, we measured the amounts of proteins adsorbed on PMEA analogous polymer substrates after immersion in 10% fetal bovine serum (FBS)-containing medium for 1 h (Fig 1A). The amount of protein adsorbed on the PTHFA substrate was approximately 20% less than that adsorbed on tissue culture polystyrene (TCPS). The amounts of proteins adsorbed on PMEA, PMe3A, and PMe2A substrates were approximately 45% less than that adsorbed on TCPS. Few proteins adsorbed on the poly(2-methacryloyloxyethyl phosphorylcholine-*co*-butyl methacrylate) (PMPC, 30:70 mol%) substrate. This result indicates that the amount of adsorbed proteins varies among different PMEA analogous polymer substrates.

In addition to the amount of adsorbed proteins, cell attachment is also influenced by the conformation of the adsorbed proteins, such as fibronectin (FN) [15]. The conformation of adsorbed fibronectin is altered, with its cell attachment site exposed to the cells, thus allowing cell attachment. Therefore, we compared the conformational change of fibronectin adsorbed on PMEA analogous polymer substrates by enzyme-linked immunosorbent assay (ELISA) (Fig 1B). Approximately twice as many cell attachment sites were observed for the human FN (hFN) adsorbed on PTHFA substrate compared to FN adsorbed on TCPS. The exposed cell attachment sites in the adsorbed hFN were gradually decreased across the PMEA, PMe3A, PMe2A, and PMPC substrates. This result indicates that the conformational change of fibronectin varies for cell attachment among different PMEA analogous polymer substrates.

### 2.2. Cell attachment profiles on PMEA analogous polymer substrates

**Fig 2:**
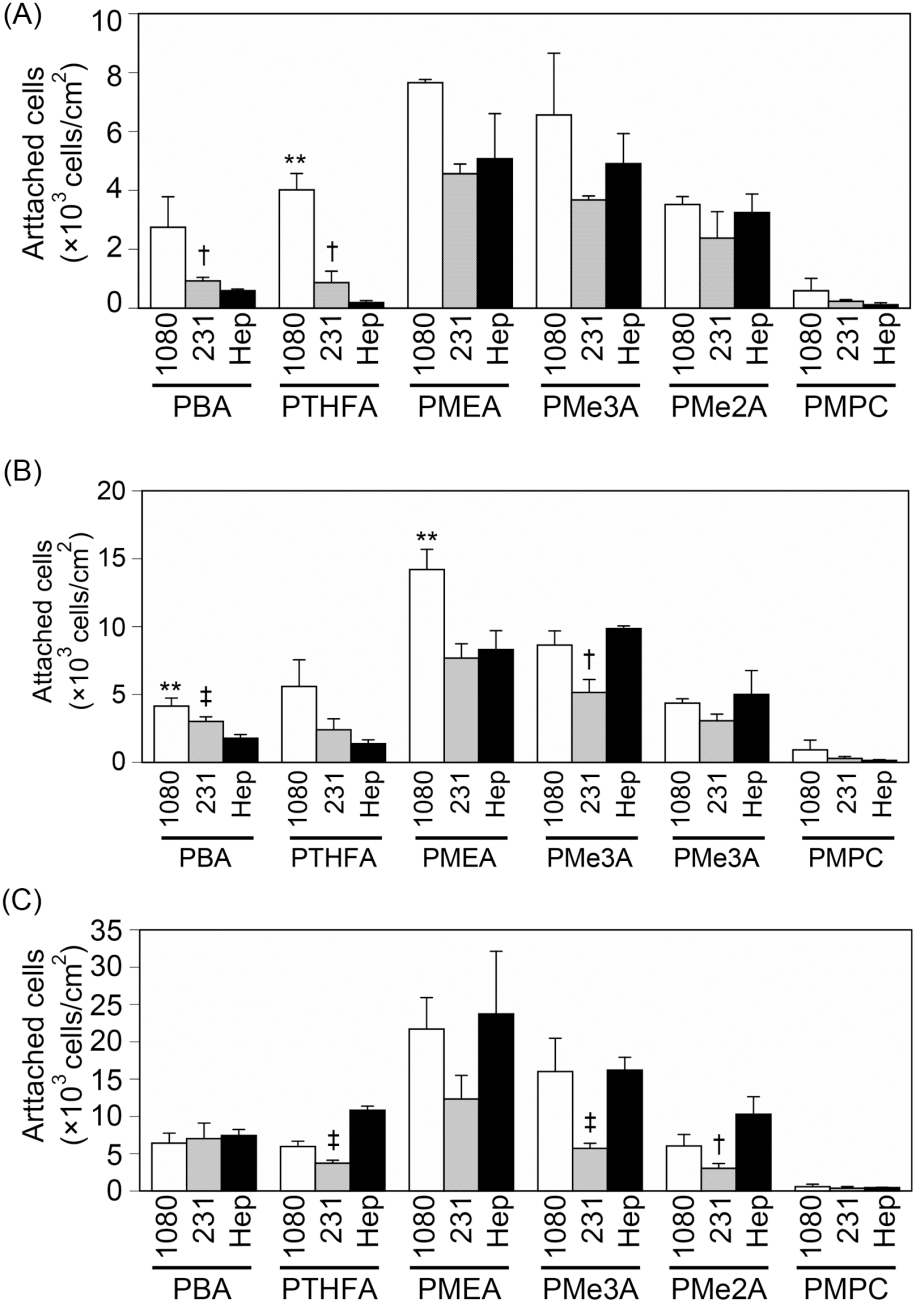
Cell attachment profiles on the PMEA-analogous polymer substrates in serum-containing medium. Cells were incubated for (A) 30 min, (B) 1 h, and (C) 3 h to allow cell attachment in serum-containing medium. 1080, 231, and Hep indicate HT-1080, MDA-MB-231, and HepG2, respectively. The data represent the means ± SD (n = 3). *: *P* < 0.05, **: *P* < 0.01, ***: *P* < 0.005 *vs*. HepG2.

Next, we examined the attachment of HT-1080 (a fibrosarcoma cell line), MDA-MB-231 (a breast tumor cell line), and HepG2 (a hepatocarcinoma cell line) cells on PMEA analogous polymer substrates in serum-containing medium after 30 min, 1 h, and 3 h to evaluate initial cell attachment profile and to examine whether there are any differences in attachment profiles among these cell types (Fig 2). HT-1080 cells started to attach to the PBA and PTHFA substrates within 30 min, whereas MAD-MB-231 and HepG2 cells did not start to attach to these substrates (Fig 2A). MDA-MB-231 cells started to attach to the PBA and PTHFA substrates after 1 h of incubation (Fig 2B). HepG2 cells were barely attached to the PBA and PTHFA substrates, even after 1 h (Fig 2B). The HepG2 cells attached to the PBA and PTHFA substrates after 3 h (Fig 2C). In contrast to the PBA and PTHFA substrates, HT-1080, MDA-MB-231, and HepG2 cells started to attach to the PMEA, PMe3A, and PMe2A substrates within 30 min (Fig 2A). On the PMPC substrate, the cells barely attached, even after 3 h. These results indicate that the cell attachment profile is dependent on both the type of cells and the PMEA analogous polymers.

### 2.3. The contribution of integrin to cell attachment on PMEA analogous polymer substrates

**Fig 3:**
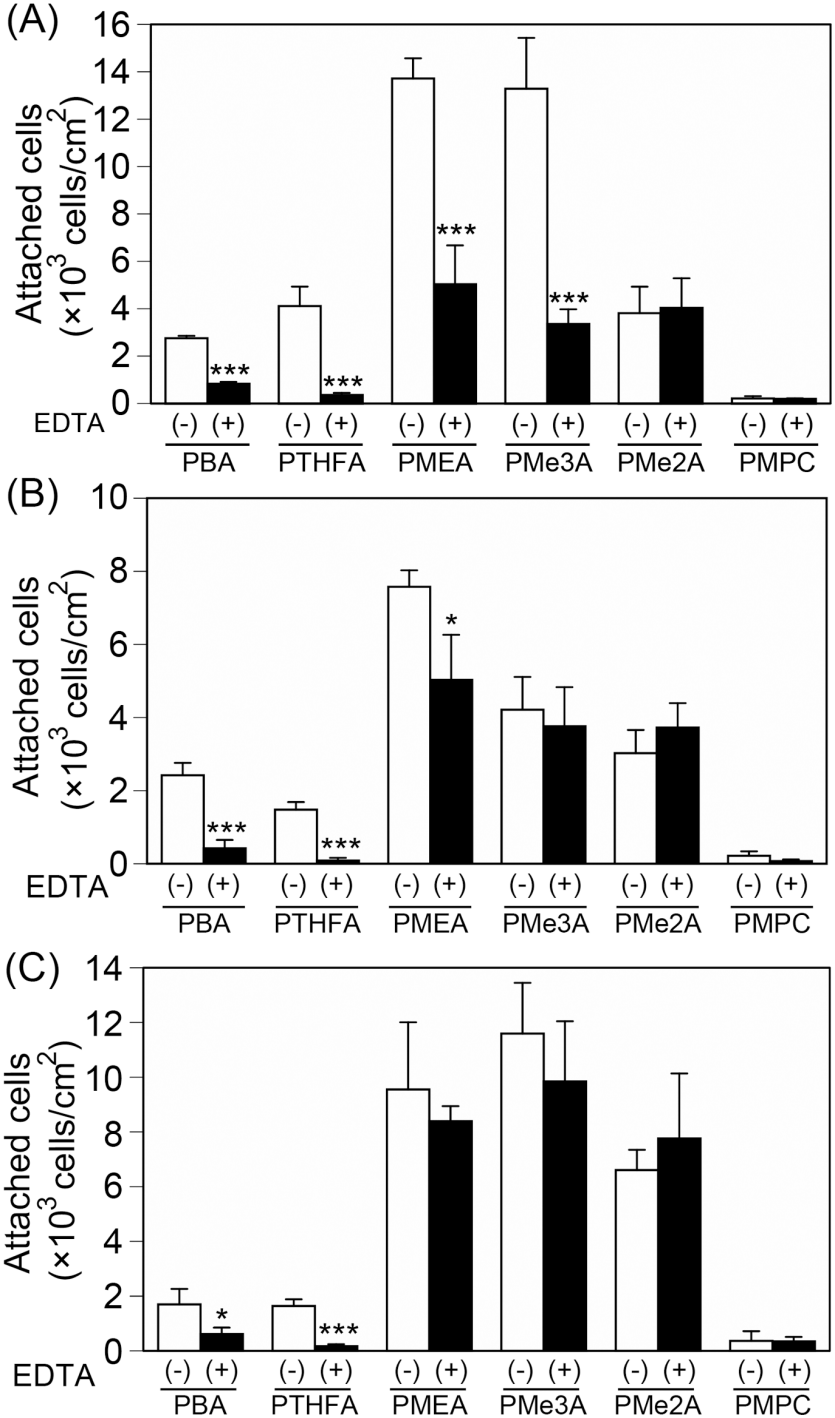
Inhibitory effect of EDTA on cell attachment. (A) HT-1080, (B) MDA-MB-231, and (C) HepG2 were incubated with PMEA-analogous polymer substrates in serum-containing medium for 1 h. The data represent the means ± SD (n = 3). *: *P* < 0.05, ***: *P* < 0.005 *vs*. EDTA (-).

We assumed that protein adsorption suppression switches the cell attachment mechanism from an integrin-dependent mechanism to an integrin-independent mechanism on PMEA analogous polymer substrates. The contribution of integrin to cell attachment on PMEA analogous polymer substrates was examined. Cell attachment was compared between these substrates in the presence of EDTA, which inhibits integrin-dependent attachment (Fig 3) [16]. EDTA decreased the number of HT-1080, MDA-MB-231, and HepG2 cells attached to the PBA and PTHFA substrates to the levels observed on the PMPC substrate, indicating that the cells attached to the PBA and PTHFA substrates via an integrin-dependent mechanism. However, the attached cells remained on the PMEA, PMe3A, and PMe2A substrates, suggesting that integrin contributions were weakened on these substrates. This observation also suggests that the cells attached to these substrates via either integrin-dependent and integrin-independent mechanisms, or both.

**Fig 4:**
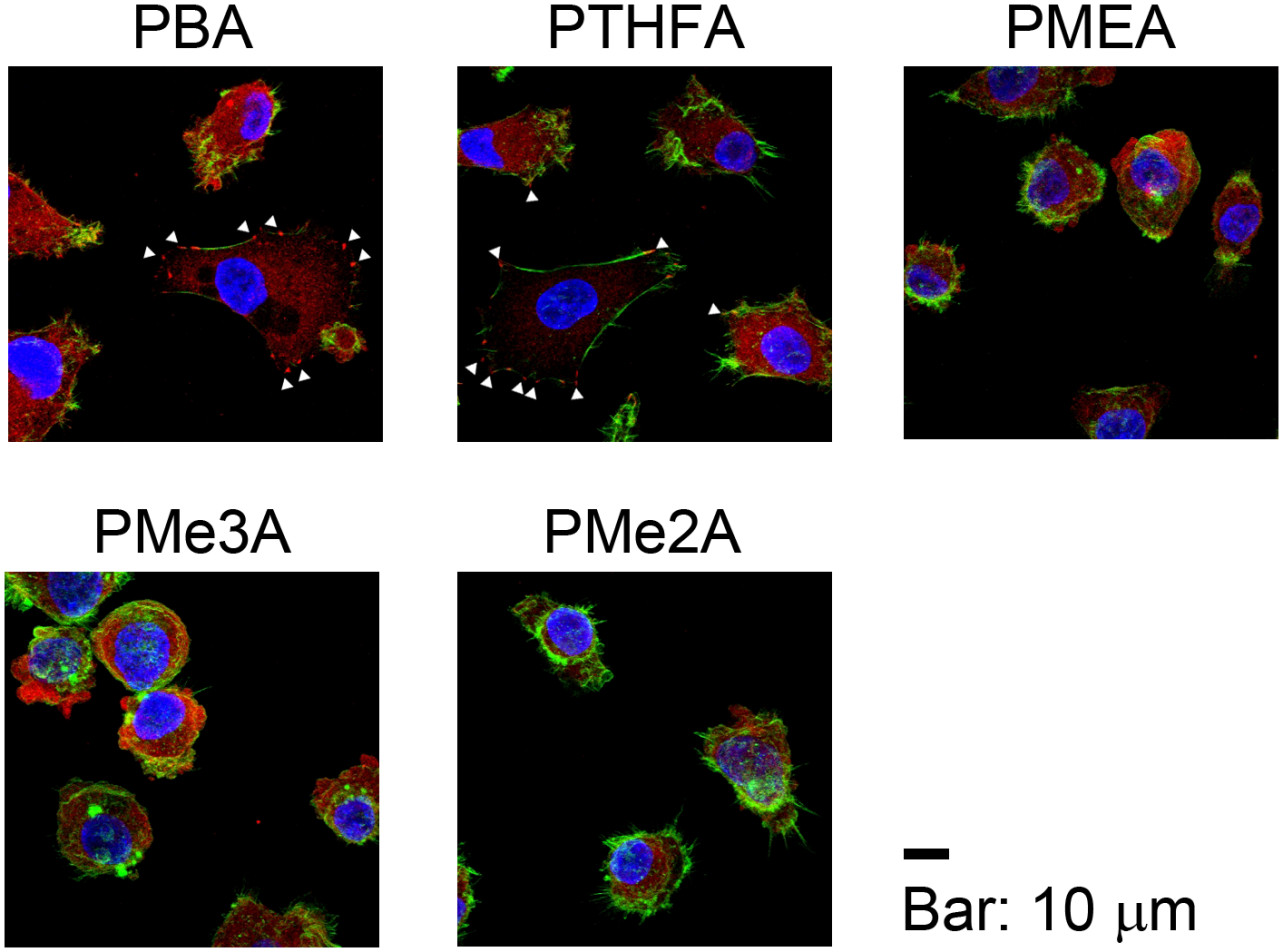
Focal adhesion formation of HT-1080 on PMEA-analogous polymer substrates after 1 day. Blue, green, and red colors depict cell nuclei, actin filaments, and vinculin, respectively. The white arrowhead indicates a focal adhesion. The bar indicates 10 μm. Focal adhesion formations in MDA-MB-231 and HepG2 are shown in Figure A and Figure B in S1 File, respectively.

For further confirmation, focal adhesion formation was also evaluated on PMEA analogous polymer substrates (Fig 4, Figures A and B in S1 File). Focal adhesions were evident in the HT-1080, MDA-MB-231, and HepG2 cells on the PBA and PTHFA substrates. In contrast, focal adhesions were observed to be minimal in these cells on the PMEA, PMe3A, and PMe2A substrates, which suggests that integrin-dependent attachment was weak on these substrates. These results indicate that the contribution of integrin to cell attachment on PMEA analogous polymer substrates is variable.

### 2.4. Attachment characteristics of HT-1080, MDA-MB-231, and HepG2 cells

**Fig 5:**
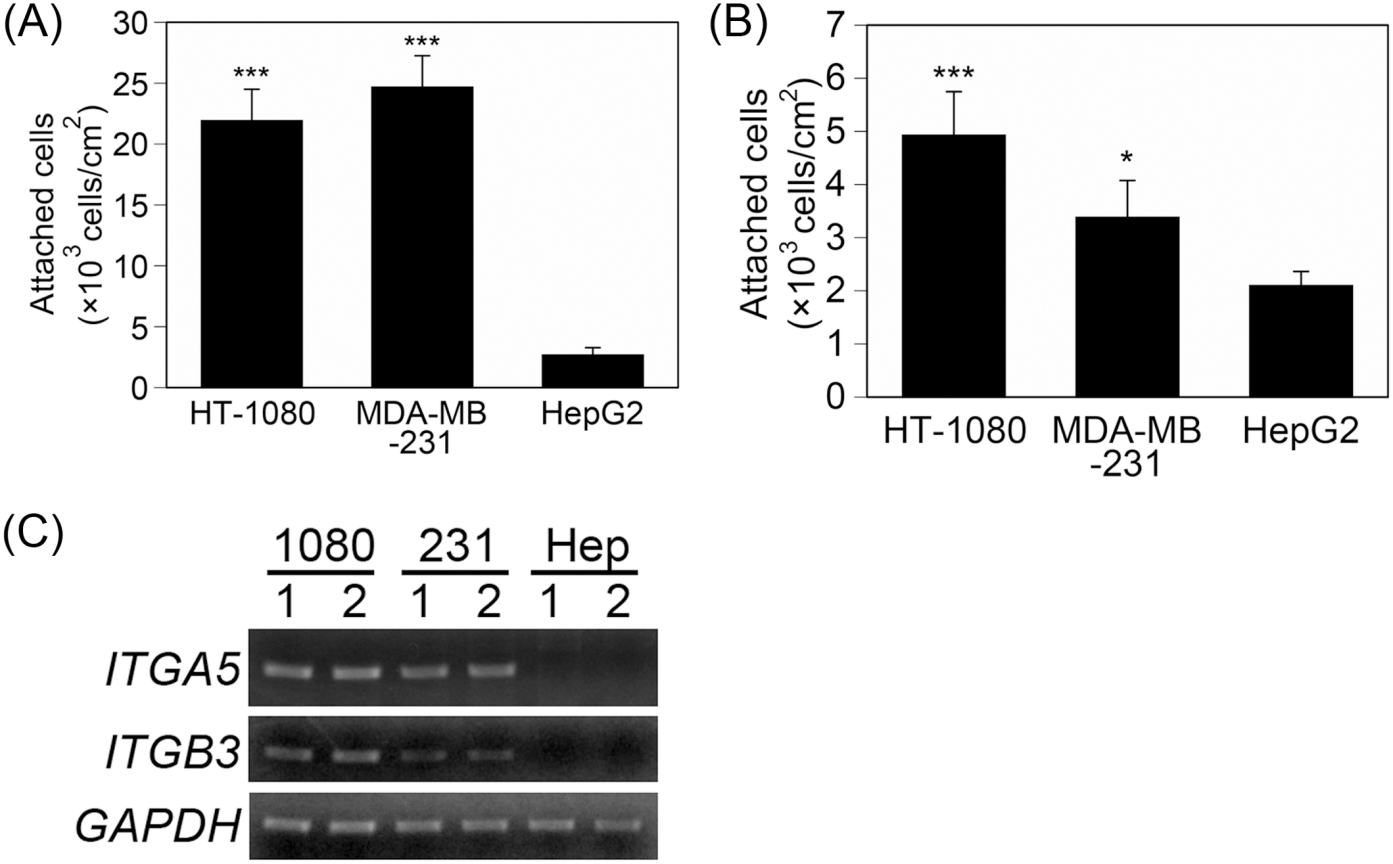
Integrin-dependent attachment. (A) Cell attachment on FN-coated substrates after 1 h in serum-free medium. (B) Cell attachment on FBS-coated substrates after 1 h in serum-free medium. The data represent the means ± SD (n = 3). *: *P* < 0.05, ***: *P* < 0.005 *vs*. HepG2. (C) Expression of integrin α5 (*ITGA5*) and β3 (*ITGB3*) chains. 1080, 231, and Hep indicate HT-1080, MDA-MB-231, and HepG2, respectively.

To investigate the differences in the cell attachment profile among HT-1080, MDA-MB-231, and HepG2 cells, we focused on the characteristics of integrin-dependent and integrin-independent attachment of these cells. We compared the characteristics of the integrin-dependent attachment of these cells using an attachment assay with an FN-coated substrate in a serum-free medium (Fig 5A). HT-1080 and MDA-MB-231 cells attached well to the FN-coated substrate after 1 h, whereas HepG2 cells barely attached to the FN-coated substrate. When cells attached in serum-containing medium, they could attach to the substrates via interaction with the adsorbed FBS proteins, including FN. Therefore, we assessed how cells attach to FBS protein-coated substrates in a serum-free medium after 1 h (Fig 5B). The number of attached cells decreased in the following order: HT-1080 > MDA-MB-231 > HepG2. Specifically, the number of attached HT-1080 cells was 2.3 times greater than that of HepG2 cells.

Generally, cells attached to FBS protein-coated substrates via integrin α5β1-FN and integrin αvβ3-vitronectin interactions [11, 17, 18]. The integrin expression levels were measured with semi-quantitative RT-PCR to gain understanding of the reason for the differences in the attachment among HT-1080, MDA-MB-231, and HepG2 cells (Fig 5C). *ITGA5* and *ITGB3* were obviously expressed in HT-1080 and MDA-MB-231 cells, whereas these genes were expressed at lower levels in HepG2 cells. These results indicate that the integrin-dependent attachments of HT-1080 and MDA-MB-231 cells were stronger than those of HepG2 cells because of the difference in integrin expression.

**Fig 6:**
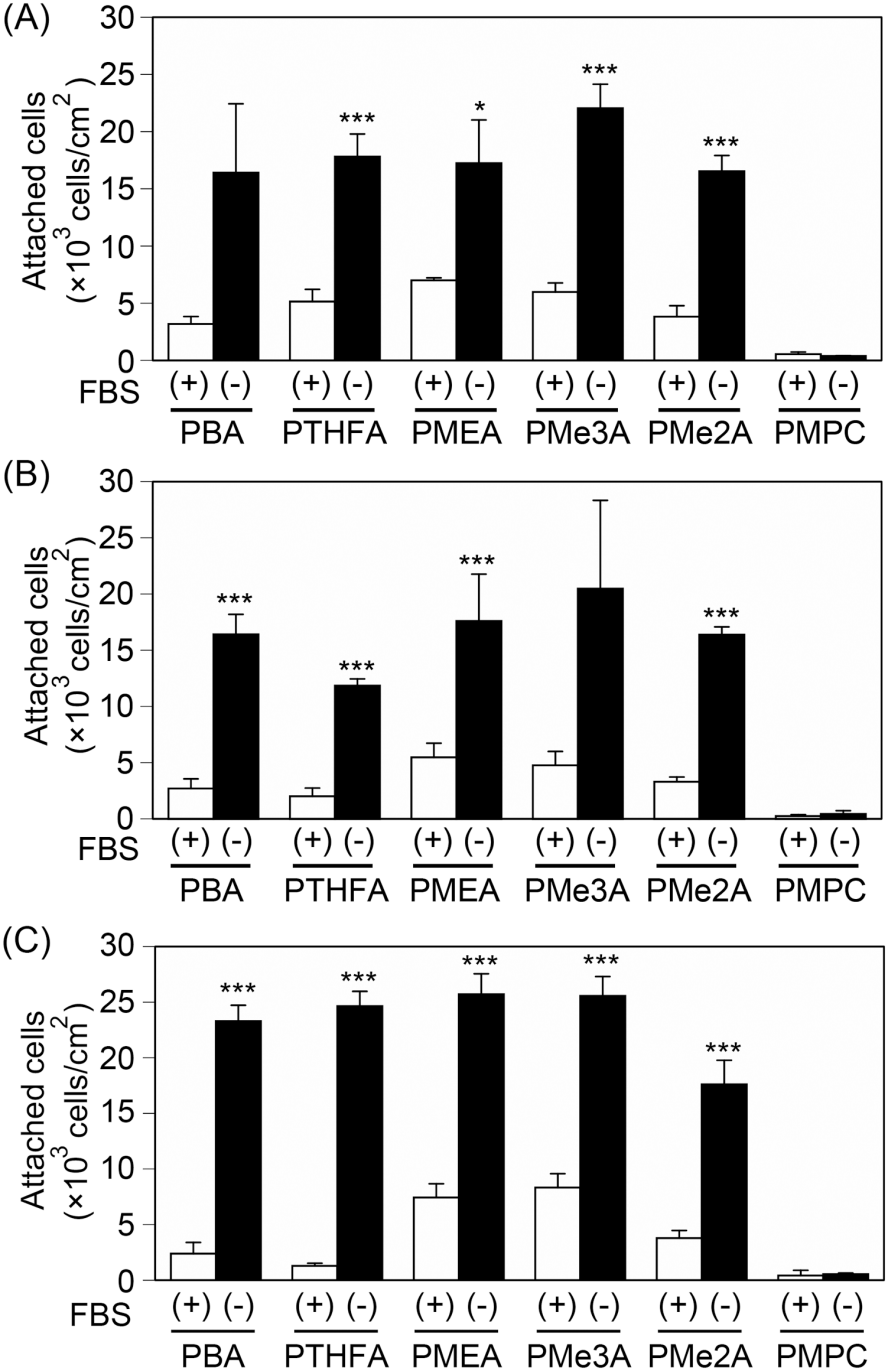
Integrin-independent attachment. (A) HT-1080, (B) MDA-MB-231, and HepG2 were incubated with the substrates in either serum-free (FBS(-)) or serum-containing (FBS(+)) media for 1 h. The data represent the means ± SD (n = 3). *: *P* < 0.05, ***: *P* < 0.005 *vs*. FBS(+).

In addition to characterizing the integrin-dependent attachment of these cells, we also compared the characteristics of integrin-independent attachment. We performed a cell attachment assay in a serum-free medium after 1 h (Fig 6). The HT-1080, MDA-MB-231, and HepG2 cells hardly attached to the PMPC substrate within 1 h in both serum-containing and serum-free media. Conversely, these cells attached to the PBA, PTHFA, PMEA, PMe3A, and PMe2A substrates even in serum-free medium. These results indicate that HT-1080, MDA-MB-231, and HepG2 cells possess the ability to attach to these polymer substrates via integrin-independent attachment without adsorbed proteins.

### 2.5. Enrichment of HT-1080 cells from a mixture with HepG2 cells through different attachment on PMEA analogous polymer substrates

**Fig 7:**
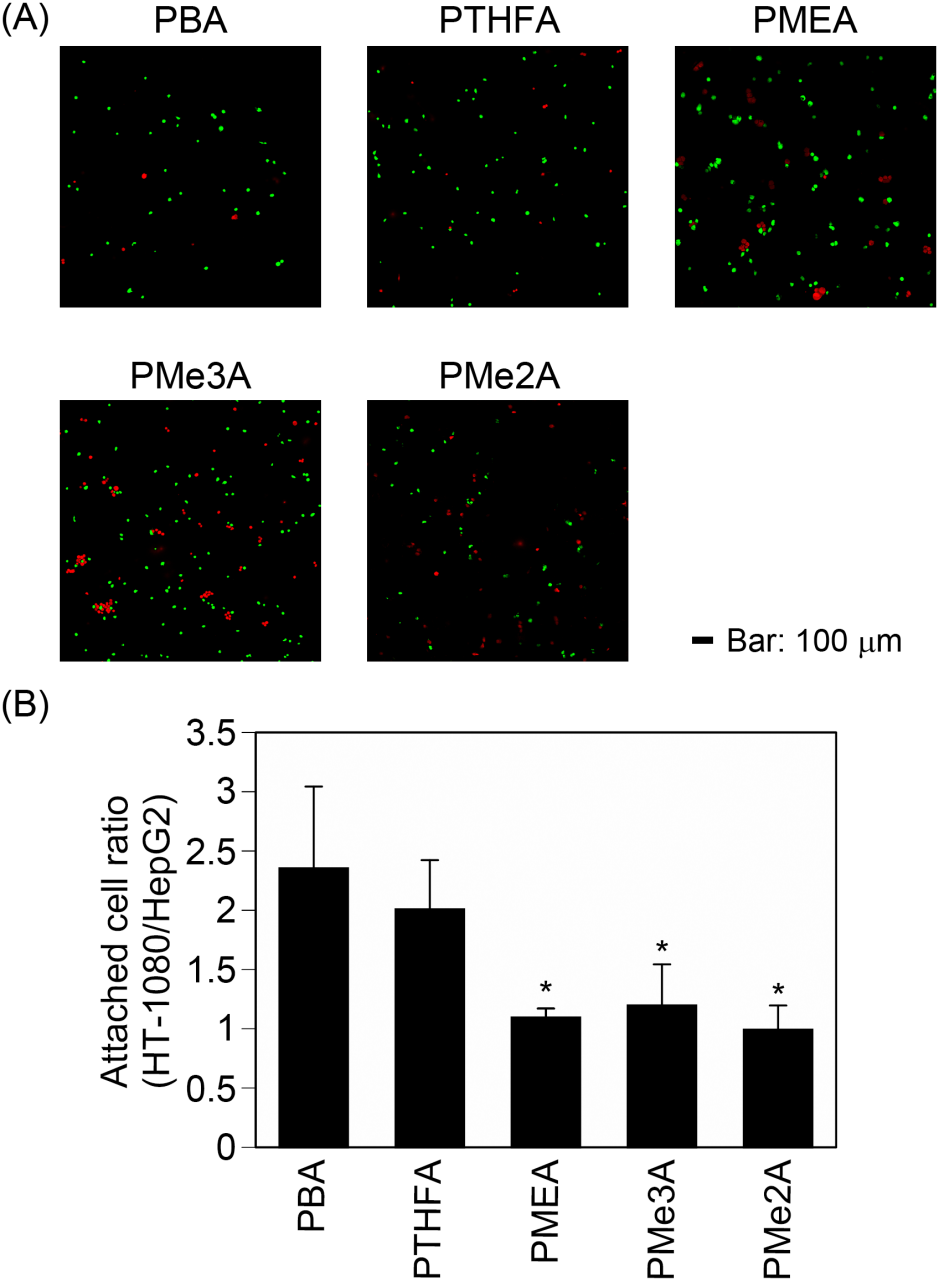
Enrichment of HT-1080 through attachment on PMEA-analogous polymer substrates. A mixture of HT-1080 and HepG2 cells (1:1) was allowed to attach to the substrates in serum-containing medium for 1 h. (A) Photographs of cells attached to the substrates. Green and red cells indicate HT-1080 and HepG2, respectively. (B) Quantitative analysis of cell enrichment. The data represent the means ± SD (n = 3). *: *P* < 0.05 *vs*. PBA and PTHFA.

Finally, we examined the possibility of cell enrichment using PMEA analogous polymer substrates depending on different cell attachment profiles. A mixture of HT-1080 and HepG2 cells (1:1) was seeded on PMEA analogous polymer substrates in serum-containing medium, and the cells were allowed to attach for 1 h. The numbers of attached HT-1080 cells on the PBA and PTHFA substrates were 2–2.4 times greater than the numbers of attached HepG2 cells on these substrates (Fig 7). In contrast, the numbers of attached HT-1080 cells on the PMEA, PMe3A, and PMe2A substrates were similar to the numbers of attached HepG2 cells on these substrates (Fig 7). These results suggested that the ratio of attached cells from a cell mixture on PMEA analogous polymer substrates can vary.

## Discussion

We demonstrated that the number of attached HT-1080 and MDA-MB-231 cells were higher than the number of attached HepG2 cells on the PBA and PTHFA substrates within 1 h (Fig 2A and 2B). The cells attached to the PBA and PTHFA substrates exclusively through an integrin-dependent mechanism (Fig 3). Generally, cells attach to polymer substrates through the interaction with FN and vitronectin adsorbed from the serum [10, 16]. Therefore, it has been suggested that the cells attached to the PBA and PTHFA substrates through the interaction with FN and vitronectin adsorbed from the serum. The HT-1080 and MDA-MB-231 cells exhibited strong integrin-dependent attachment because they expressed integrin α5β1 and αvβ3, which are major receptors against FN and vitronectin [17] (Fig 5C). In contrast, HepG2 cells expressed these integrins at a very low level, which causes weaker integrin-dependent attachment to FN and vitronectin (Fig 5C). Therefore, the HepG2 cells weakly attached to FN and vitronectin adsorbed on the PBA and PTHFA substrates through the minor integrin receptors, such as integrin α4β1, αvβ1, and αvβ6, exhibiting lower cell attachment on these substrates compared to that of HT-1080 and MDA-MB-231 cells.

Conversely, the cells attached to the PMEA, PMe3A, and PMe2A substrates through both integrin-dependent and integrin-independent mechanisms (Fig 3). The amount of protein adsorption on the PMEA, PMe2A, and PMe3A substrates was significantly lower than the adsorption observed on the PBA and PTHFA substrates (Fig 1A), suggesting that the substrate surface was exposed to the cells. The amount of adsorbed proteins on PMEA was approximately 200 ng/cm^2^, which was expected to expose the bare polymer surface [13]. Therefore, it is also expected that the bare surface of PMe3A and PMe2A substrates was exposed, even in serum-containing medium. The exposed surface can directly interact with the cells, and the cells can attach to the substrates even in the absence of serum proteins (Fig 6). In serum-free medium, few proteins are adsorbed on the substrates and the bare surfaces may be exposed to the cells. Therefore, integrin-independent attachment might be involved in physical interactions, such as a hydrophobic interaction between bare polymer substrates and the proteins on the cell membrane. Integrin-independent attachment mechanism should be clarified in future studies.

The numbers of attached HT-1080, MDA-MB-231, and HepG2 cells were higher in serum-free medium than in serum-containing medium (Fig 6), suggesting that integrin-independent attachment of these cells occurred more rapidly than integrin-dependent attachment. Therefore, the cells rapidly attached to the PMEA, PMe3A, and PMe2A substrates, for which the contribution of integrin to cell attachment is low. Additionally, the numbers of attached cells were similar in serum-free medium among HT-1080, MDA-MB-231, and HepG2 cells. HT-1080, MDA-MB-231, and HepG2 cells attached to the PMEA, PMe3A, and PMe2A substrates via both integrin-dependent and integrin-independent mechanisms. The integrin-independent attachment of these cells occurred more rapidly than the integrin-dependent attachment. Therefore, the number of attached cells on the PMEA, PMe3A, and PMe2A substrates was higher relative to the number of cells that attached to the PBA and PTHFA substrates.

We demonstrated and discussed that the contribution of integrin to cell attachment can be changed by the regulation of protein adsorption on PMEA analogous polymer substrates. We have previously reported that a hydrated condition of polymers strongly influenced protein adsorption [13, 19–21]. A unique hydrated structure called intermediate water was observed in hydrated PMEA analogous polymers [19–20]. Intermediate water functioned as a barrier against the interaction between proteins and the substrate surface [21]. Additionally intermediate water suppresses protein adsorption on the substrate surface [13, 21]. Intermediate water contents in the hydrated PMEA analogous polymers used in this study are different and are in the order of PBA < PTHFA < PMEA < PMe3A < PMe2A < PMPC (Table A in S1 File) [14, 22, 23]. When the amounts of protein adsorption were compared among PMEA analogous polymer substrates, the amount decreased with the increase of intermediate water content (Fig 1A and Figure C in S1 File). This result suggests that the amount of protein adsorption and the contribution of integrin to cell attachment can be regulated by the substrates coated with PMEA analogous polymers with different intermediate water contents (Figure D in S1 File).

We have previously shown that the type of a specific protein (*e*.*g*., albumin and fibrinogen) influences the degrees to which the intermediate water molecules suppressed protein adsorption [19]. The amount of adsorbed FBS proteins was similar between PMEA and PMe3A/PMe2A, although there is a large difference on intermediate water contents between PMEA and PMe3A/PMe2A. Proteins with easily suppressed adsorption with low content of intermediate water were suppressed on PMEA, PMe3A and PMe2A. In contrast, proteins whose adsorption is barely suppressed with low and medium content of intermediate water were not suppressed on these polymers. Therefore, the amounts of adsorbed FBS proteins are similar on PMEA, PMe3A, and PMe2A.

We demonstrated that the ratio of the cell mixture can be changed via attachment on PMEA analogous polymer substrates (Fig 7). Although the attachment of HT-1080 cells was approximately 1.5 times higher than that of HepG2 on PMEA, PMe3A, and PMe2A (Fig 2B), the ratio of the attached cells from the cell mixture was similar between HT-1080 and HepG2 cells on these polymers (Fig 7B). It has been reported that cadherin-mediated cell-cell attachment influenced cell-ECM attachment via integrin [24]. In the cell mixture experiment (Fig 7), the cell densities of HT-1080 and HepG2 were half of the condition of cell attachment assays (Fig 2). Therefore, the differences of cell density might have influenced the cell attachment behaviors shown in Figs 2 and 7 to change the cell attachment profiles and the cell attachment ratio on PMEA, PMe3A, and PMe2A.

**Fig 8:**
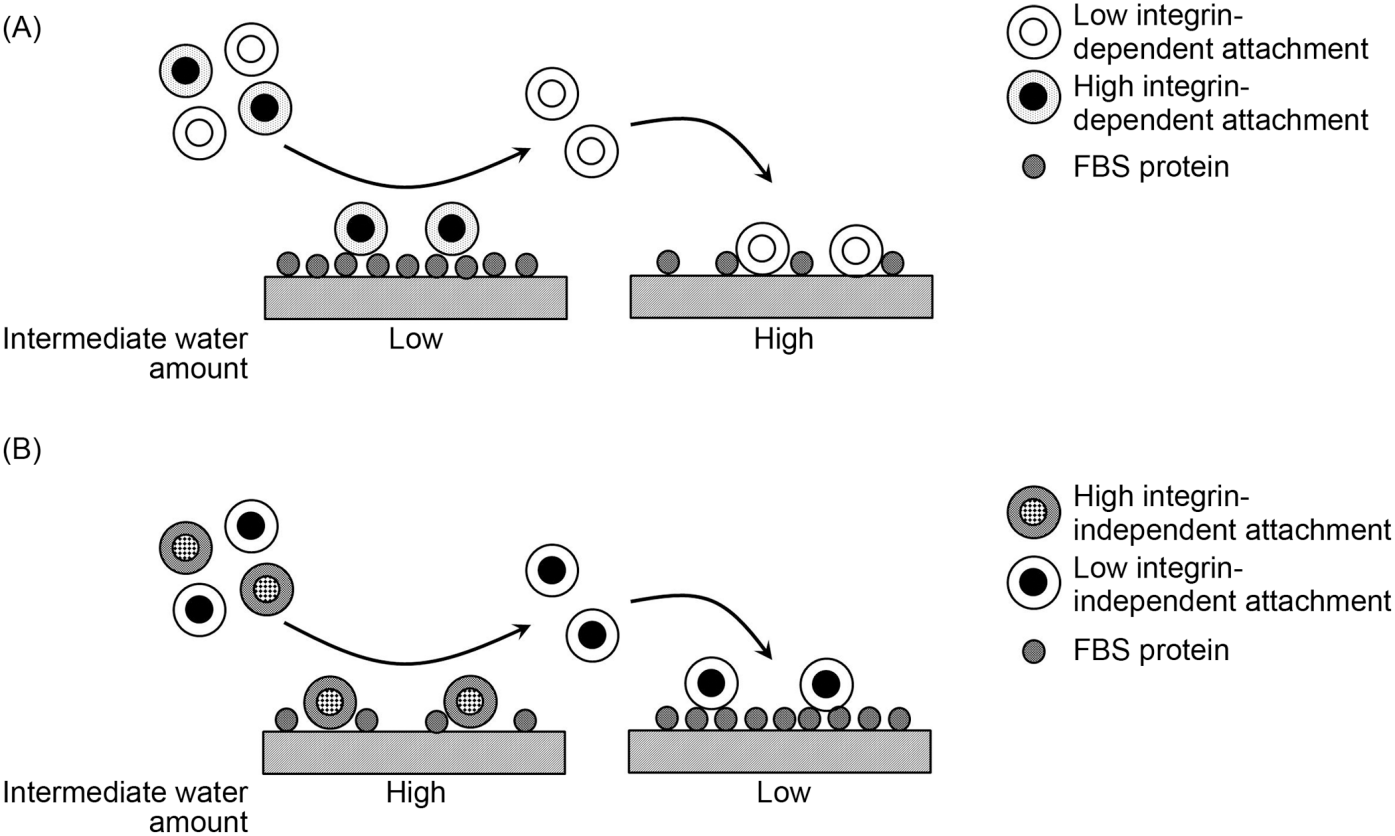
Possibility of cell enrichment via regulation of protein adsorption using PMEA-analogous polymer substrates with different intermediate water contents. (A) Enrichment of cells with various integrin-dependencies for attachment (*i*.*e*., a dependency on the adsorbed protein-mediated attachment). (B) Enrichment of cells with various integrin-independent types of attachment (*i*.*e*., a direct interaction with the polymer substrates).

Cell attachment on PMEA analogous polymer substrates represents a potential application for attachment-based enrichment. For attachment-based enrichment, fine-tuning of cell attachment strength can expand its application to various types of cells. We observed that intermediate water can change the contribution of integrin to cell attachment (*i*.*e*., cell attachment strength). Therefore, it is expected that the substrates coated with PMEA analogous polymers with different intermediate water contents can expand the attachment-based enrichment for a variety of cells (Fig 8). However, future studies should confirm if the cells that exhibit low integrin-independent attachment can be enriched from a mixture with cells that exhibit high integrin-independent attachment (Fig 8B).

As discussed above, differences in cell attachment on PMEA analogous polymer substrates appeared to be due to the differences in integrin expression. Integrin expression levels depend on tissue type and on the cellular state during developmental stages [25]. For example, MSCs retain integrin α5β1 expression during osteogenic processes, whereas they lose this expression during adipogenic processes [25]. Moreover, the expression levels of integrins change when matured cells are dedifferentiated [26, 27]. The expression of integrin α5β1 in chondrocytes increases during the progression of dedifferentiation [26]. Attachment-based enrichment using PMEA analogous polymer substrates can be applied to enrich MSC-derived matured cells and chondrocytes with high cartilage-specific activities for regenerative medicine, as well as in the quality control of cultured cells. Moreover, PMEA analogous polymer substrates also exhibit blood compatibility [14, 22, 27]. Therefore, these substrates can be used to enrich cells (*e*.*g*., CTCs and EPCs), even in the presence of blood.

In the present study, cell attachment was performed under static conditions. Fluidic conditions, such as microfluidic systems and cell rolling columns, are expected to amplify the variation in cell attachment [28, 29]. Applying fluidic conditions is one way to improve cell enrichment efficiency. Compared with antibody-based cell enrichment, our method does not require any well-trained operators or special apparatuses. Additionally, cells enriched by our method are free from antibody-labeling, which is desirable for clinical applications.

In conclusion, PMEA analogous polymer substrates can regulate protein adsorption behaviors on various substrates. PMEA analogous polymer substrates affect the contribution of integrin to the attachment to the substrates via the regulation of protein adsorption. Changes in the contribution of integrin to the attachment of cells to the substrates lead to different cell attachment profiles, due to different integrin expression levels. Therefore, PMEA analogous polymer substrates can be applied for attachment-based enrichment of cells.

## Materials and Methods

### 4.1. Preparation of polymer substrates

PMEA (Mw: 150,000 g/mol) and PTHFA (Mw: 150,000 g/mol) were synthesized as previously described [22, 27]. Poly(butyl acrylate) (PBA, Mw: 72,700 g/mol), poly(2-(2-methoxyethoxy) ethyl acrylate-*co*-butyl acrylate) (30:70 mol%, PMe2A, Mw: 40,500 g/mol) and poly(2-(2-methoxyethoxy) ethoxy ethyl acrylate-*co*-butyl acrylate) (30:70 mol%, PMe3A, Mw: 35,600 g/mol) were also synthesized as described in a previous report [14]. Poly(2-methacryloyloxyethyl phosphorylcholine-*co*-butyl methacrylate) (30:70 mol%, PMPC, Mw: 600,000 g/mol) was a generous gift from the NOF Corporation (Tokyo, Japan). The chemical structures of these polymers are shown in Figure E in S1 File.

The polymer substrates were prepared on polyethylene terephthalate (PET) discs (ϕ = 14 mm, thickness = 125 μm, Mitsubishi Plastics, Tokyo, Japan). Briefly, PMEA, PMe2A, PMe3A, and PMPC were dissolved in methanol at a concentration of 0.2% (w/v). PTHFA was dissolved in methanol/chloroform (5:1) at a concentration of 0.2% (w/v). Forty milliliters of each polymer solution was cast on the PET disc and spin-coated twice under the following conditions: 500 rpm for 5 sec, 2,000 rpm for 10 sec, 2,000 to 4,000 rpm (slope) for 5 sec, 4,000 rpm for 10 sec, and 4,000 to 0 rpm (slope) for 5 sec. The polymer substrates were sterilized by UV exposure for 2 h and stored at 4°C until further use. Bovine plasma-derived fibronectin (FN, Calbiochem, Darmstadt, Germany)-coated substrates were prepared on PET discs. Sterilized PET discs were immersed in the FN solution (10 μg/mL) for 4 h at 37°C for protein coating. After coating, the discs were washed with water and air-dried for 1 h. Fetal bovine serum (FBS, Equitech-Bio, Kerrville, TX)-coated substrates were prepared by immersion in FBS-containing Dulbecco’s Modified Eagle/Nutrient Mixture F-12 (DMEM/F-12, Gibco, Carlsbad, CA) for 1 h. The prepared substrates were washed twice with phosphate-buffered saline (PBS) and then immediately used.

Polymer-cast substrates were prepared to quantify adsorbed FBS proteins and to perform enzyme-linked immunosorbent assays (ELISAs). Briefly, 12 μL of each polymer solution was added to a 96-well tissue culture polystyrene (TCPS) plate (IWAKI, Tokyo, Japan), and the plate was air-dried for 1 week. Instead of PBA, a bare TCPS surface was used as the substrate without intermediate water.

### 4.2. Quantification of adsorbed FBS proteins on the substrates

Polymer-cast 96-well TCPS plates were immersed in PBS for 1 h at 37°C. Subsequently, 100 μL of 10% FBS-containing DMEM/F-12 medium (serum medium) was added to each well. After 1 h of incubation at 37°C, the incubated wells were washed seven times with PBS. The adsorbed proteins were extracted by incubating the plate with a 5% sodium dodecyl sulfate (SDS) solution and 0.1 N NaOH for 60 min at room temperature. The extracted proteins were assessed with a microBCA assay (Thermo Scientific, Rockford, IL) according to the manufacturer’s instructions. The amount of protein was calculated using a γ-globulin standard curve.

### 4.3. Enzyme-linked immunosorbent assay (ELISA)

The polymer-cast 96-well TCPS plates were immersed in PBS for 1 h at 37°C. After incubation, human fibronectin (hFN, 5 μg/mL, 50 μL/well, Sigma) was added to the plate, and the plate was incubated for 1 h at 37°C. After the adsorption of human fibronectin, the samples were incubated with Blocking-One solution (Nacalai Tesque, Kyoto, Japan) for 30 min at room temperature to prevent non-specific reactions. After blocking, the samples were incubated with HFN7.1 antibody (Ab) (Abcam, Cambridge, UK) for 2 h at room temperature, followed by incubation with a peroxidase-conjugated anti-mouse IgG Ab for 1 h at room temperature. After these incubations, the samples were incubated with 2,2'-azinobis(3-ethylbenzothiazoline-6-sulfonic acid ammonium salt) (ABTS) substrate (Roche Diagnostics). The absorbance was measured at a wavelength of 405 nm.

### 4.4. Cell culture

The MDA-MB-231 cells were obtained from the American Type Culture Collection (ATCC, Manassas, VA). The HT-1080 and HepG2 cells were obtained from the Japanese Collection of Research Bioresources Cell Bank (JCRB Cell Bank, Osaka, Japan). All cells were maintained in DMEM/F-12 containing 10% FBS on tissue culture polystyrene (TCPS). Prior to the experiments, the cells were detached from the TCPS with a 0.25% trypsin/EDTA solution (Gibco).

### 4.5. Cell attachment assay

The polymer substrates were immersed in DMEM/F-12 medium containing 10% FBS or in serum-free DMEM/F-12 for 1 h at 37°C prior to cell culture. The cells were seeded onto the polymer substrates at a density of 5 × 10^4^ cells/cm^2^. The cells were allowed to attach to the substrates in DMEM/F-12 medium containing 10% FBS or in serum-free DMEM/F-12 for the indicated times. The non-attached cells were removed by washing the plates twice with PBS. The attached cells were fixed with 0.1% glutaraldehyde overnight at room temperature. The cells were stained with a 0.2% crystal violet (Wako, Osaka, Japan) solution for 15 min for visualization. After staining, the attached cells in three randomly selected fields were counted using an optical microscope. For the inhibition assay, the cells were treated with 5 mM EDTA for 10 min at 37°C prior to seeding of the cells. The cell attachment assay was performed after EDTA treatment, as described above.

### 4.6. Focal adhesion observation

The cells were cultured on the substrates for 1 day. After culture, the cells were fixed with 4% paraformaldehyde in PBS (Wako) for 10 min at 37°C and treated three times with 1% Triton X-100 in PBS for 10 min at room temperature. After permeabilization, the samples were incubated with an anti-vinculin Ab (Millipore, Billerica, MA) for 2 h at 37°C, followed by treatment with Alexa Fluor 488-conjugated phalloidin (Invitrogen, Carlsbad, CA) and Alexa Fluor 568-conjugated anti-mouse IgG Ab (Invitrogen) for 1 h at 37°C. Can Get Signal (ToYoBo, Osaka, Japan) was used for the observation. ProLong Gold Antifade Reagent with DAPI (Invitrogen) was used to mount the slides and to counterstain the cell nuclei, respectively. The observations were made using a confocal laser scanning microscope (Olympus, Tokyo, Japan).

### 4.7. Semi-quantitative reverse transcription-polymerase chain reaction (RT-PCR)

Total RNA was extracted from the cells cultured in serum-containing medium for 4 days using a Sepasol-RNA I Super reagent according to the manufacturer’s instructions (Nacalai Tesque, Kyoto, Japan). The total RNA (1 μg) was used as a first strand and mixed with random hexamer primers and ReverTra Ace-α reverse transcriptase (TOYOBO, Osaka, Japan). Semi-quantitative RT-PCR was performed using HybriPol DNA polymerase (Nippon Genetics, Tokyo, Japan) with specific primer sets, as shown in Table 1. All primers were obtained from Nihon Gene Research Laboratories (Sendai, Japan). *GAPDH* was amplified to normalize the expression of the genes of interest in the sample for each experiment. The PCR products were analyzed via 1% agarose gel electrophoresis.

**Table 1:** Primer sequences for semi-quantitative RT-PCR analysis

mRNA		Oligonucleotide
*GAPDH*	Forward	5’- GGGCTGCTTTTAACTCTGGT-3’
	Reverse	5’- TGGCAGGTTTTTCTAGACGG-3’
*ITGA5*	Forward	5’- GCAAGAGCCGGATAGAGGAC-3’
	Reverse	5’-GGACTGTAAACCGAAGGCCA-3’
*ITGB3*	Forward	5’-CTGCCGTGACGAGATTGAGT-3’
	Reverse	5’-ATTAAGTGCCCCGGTACGTG-3’

*GAPDH* was designed according to Tuli et al. [30]. *ITGA5* and *ITGB3* were designed in our laboratory.

### 4.8. Cell enrichment test

HT-1080 and HepG2 cells were labeled via incubation with 10 μM CellTracker Green (Life Technologies, Carlsbad, CA) and CellTracker Orange (Life Technologies) for 30 min at 37°C. After washing, equal amounts of HT-1080 and HepG2 cells were mixed and then seeded at a total cell density of 5 × 10^4^ cells/cm^2^. The non-attached cells were removed from the culture by washing twice with PBS after 1 h. The attached cells were fixed with 4% paraformaldehyde for 10 min at 37°C. The attached cells were counted in three randomly selected fields using a confocal laser scanning microscope.

### 4.9. Statistical analysis

All data are expressed as the means ± SD. The significance of the differences between two samples was determined by an unpaired Student’s *t*-test using Microsoft Excel 2010. Differences with *P* < 0.05 were considered to be statistically significant.

## Supporting Information

S1 File Supporting figures and table. (Table A) Water content in hydrated PMEA-analogous polymers (wt%). (Figure A) Focal adhesion formation of MDA-MB-231 on PMEA-analogous polymer substrates after 1 day. (Figure B) Focal adhesion formation of HepG2 on the PMEA-analogous polymer substrates after 1 day. (Figure C) Relationship between intermediate water content and protein adsorption. (Figure D) Relationship between intermediate water contents and cell attachment. (Figure E) Chemical structure of PMEA analogous polymers.
(DOC)
