# Supplementary material for: Regulation of the Contribution of Integrin to Cell Attachment on Poly(2-Methoxyethyl Acrylate) (PMEA) Analogous Polymers for Attachment-Based Cell Enrichment
Source: PLoS One. 2015 Aug 19;10(8):e0136066. doi: 10.1371/journal.pone.0136066 (PMC4545787; doi:10.1371/journal.pone.0136066)

**Supplemental Information**

Table A: Water contents in hydrated PMEA-analogous polymers (wt%) ^[1]^

Supplemental Table 1: Water contents in hydrated PMEA-analogous polymers (wt%) [21]

| Description | PBA | PTHFA | PMEA | PMe3A | PMe2A | PMPC |
| --- | --- | --- | --- | --- | --- | --- |
| Bound water | 0.68 | 4.1 | 6.3 | 42 | 36 | 55 |
| Intermediate water | 0 | 1.3 | 3.8 | 20 | 21 | 29 |
| Non-freezing water | 0.68 | 2.8 | 2.5 | 22 | 15 | 26 |

The content of water in the homopolymers of 2-(2-methoxyethoxy) ethoxy ethyl acrylate, 2-methacryloyloxyethyl phosphorylcholine, and 2-methacryloyloxyethyl phosphorylcholine are shown as the contents of water in hydrated PMe3A, PMe2A, and PMPC, respectively.

Figure A

Focal adhesion formation of MDA-MB-231 on PMEA-analogous polymer substrates after 1 day. Blue, green, and red colors depict cell nuclei, actin filaments, and vinculin, respectively. The white arrow head indicates a focal adhesion. The bar indicates 10 μm.


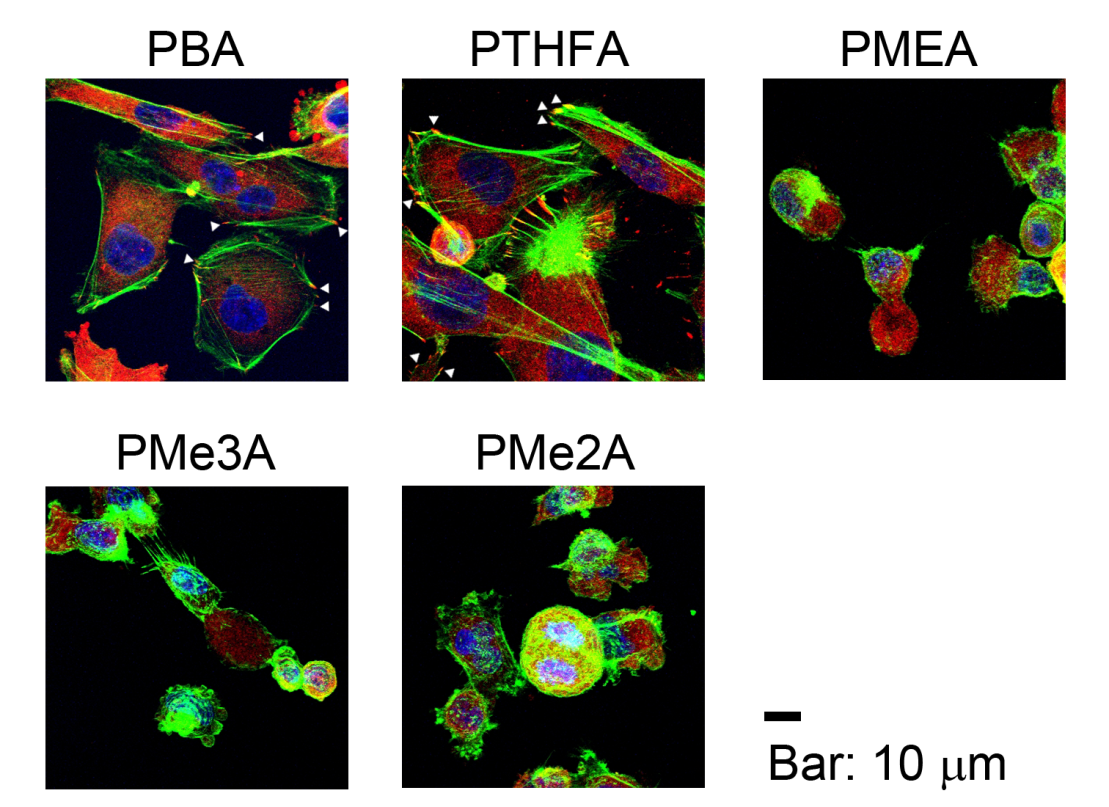


Figure B

Focal adhesion formation of HepG2 on the PMEA-analogous polymer substrates after 1 day. Blue, green, and red colors depict cell nuclei, actin filaments, and vinculin, respectively. The white arrowhead indicates a focal adhesion. The bar indicates 10 μm.


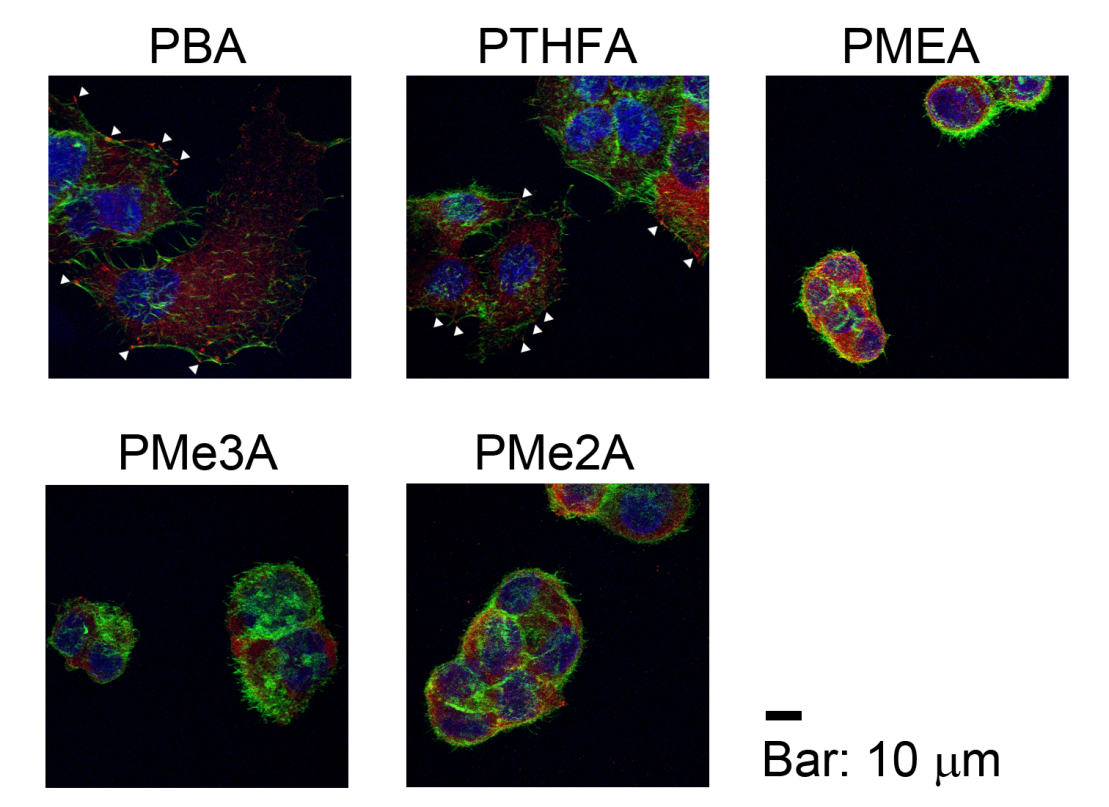


Figure C

Relationship between intermediate water content and protein adsorption. (A) Relationship between intermediate water content and the amounts of adsorbed FBS proteins. The data represent the means ± SD (n=5). (B) Relationship between intermediate water content and exposed cell attachment site in FN. The data represent the means ± SD (n=4).


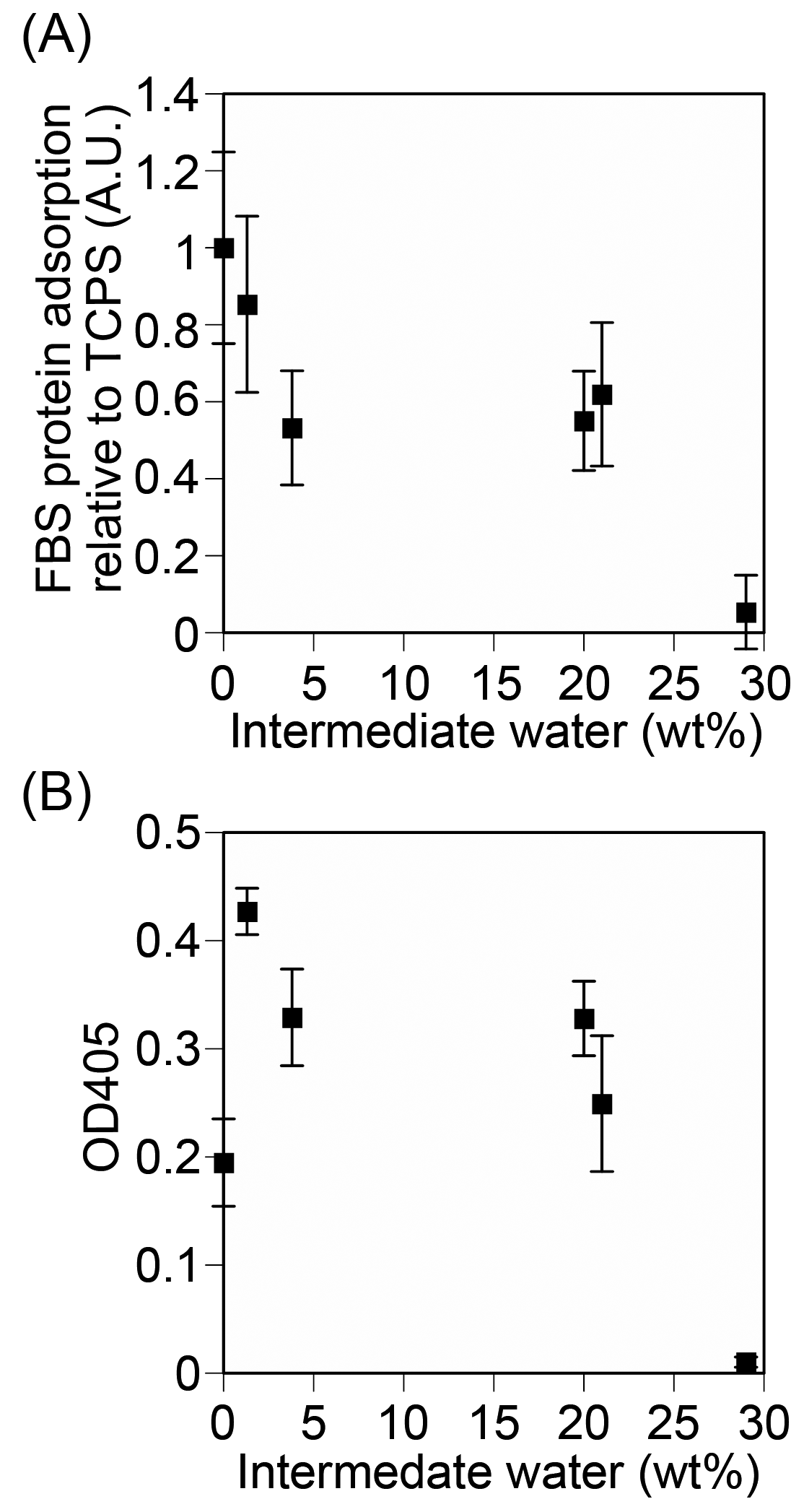


Figure D

Relationship between intermediate water contents and cell attachment after incubation for (A) 30 min, (B) 60 min, and (C) 180 min. The square and circle plots indicate HT-1080 and HepG2, respectively. The shadowed window indicates intermediate water contents that showed high HT-1080 attachment and low HepG2 attachment.


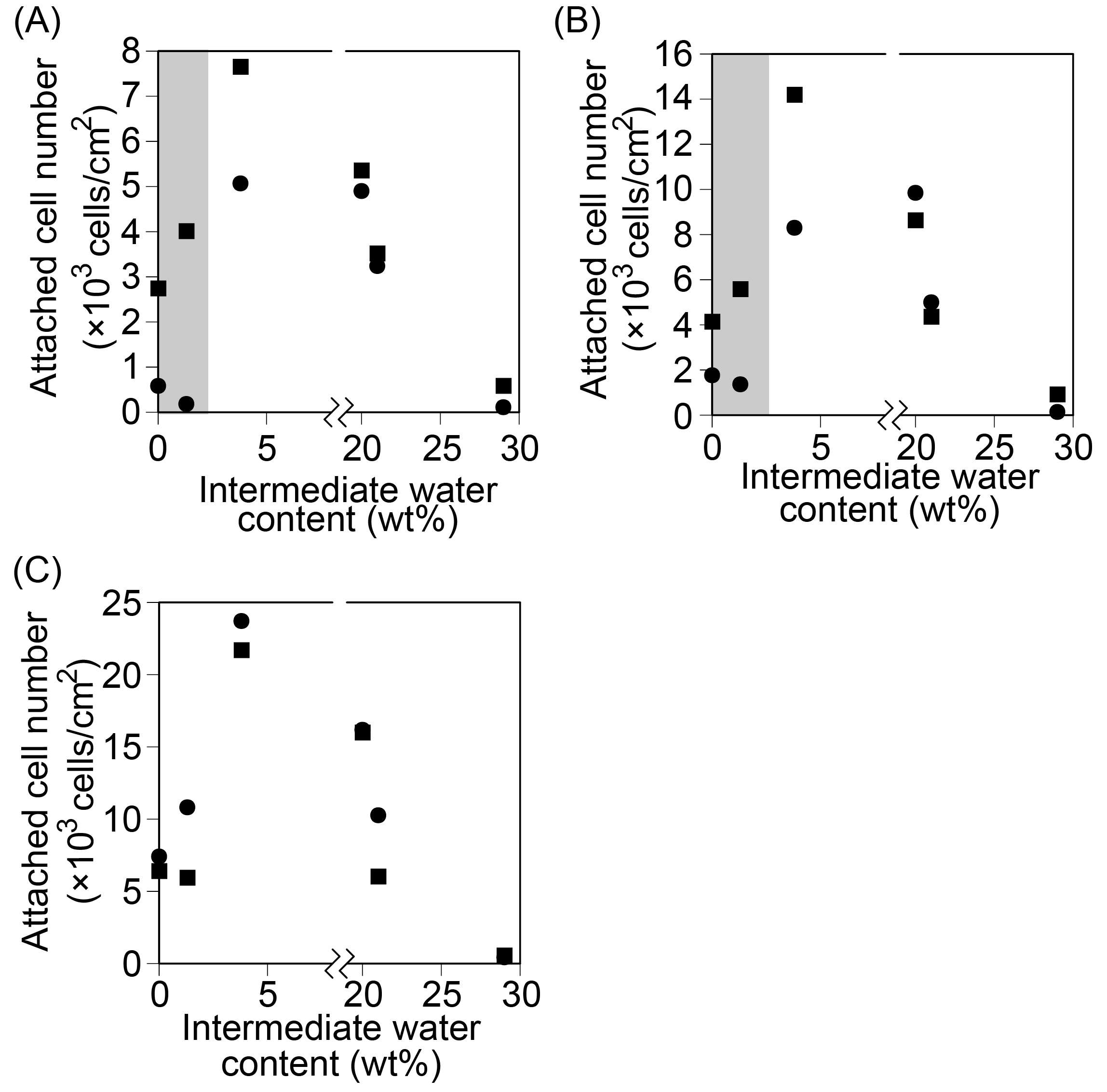


Figure E

Chemical structure of PMEA analogous polymers.


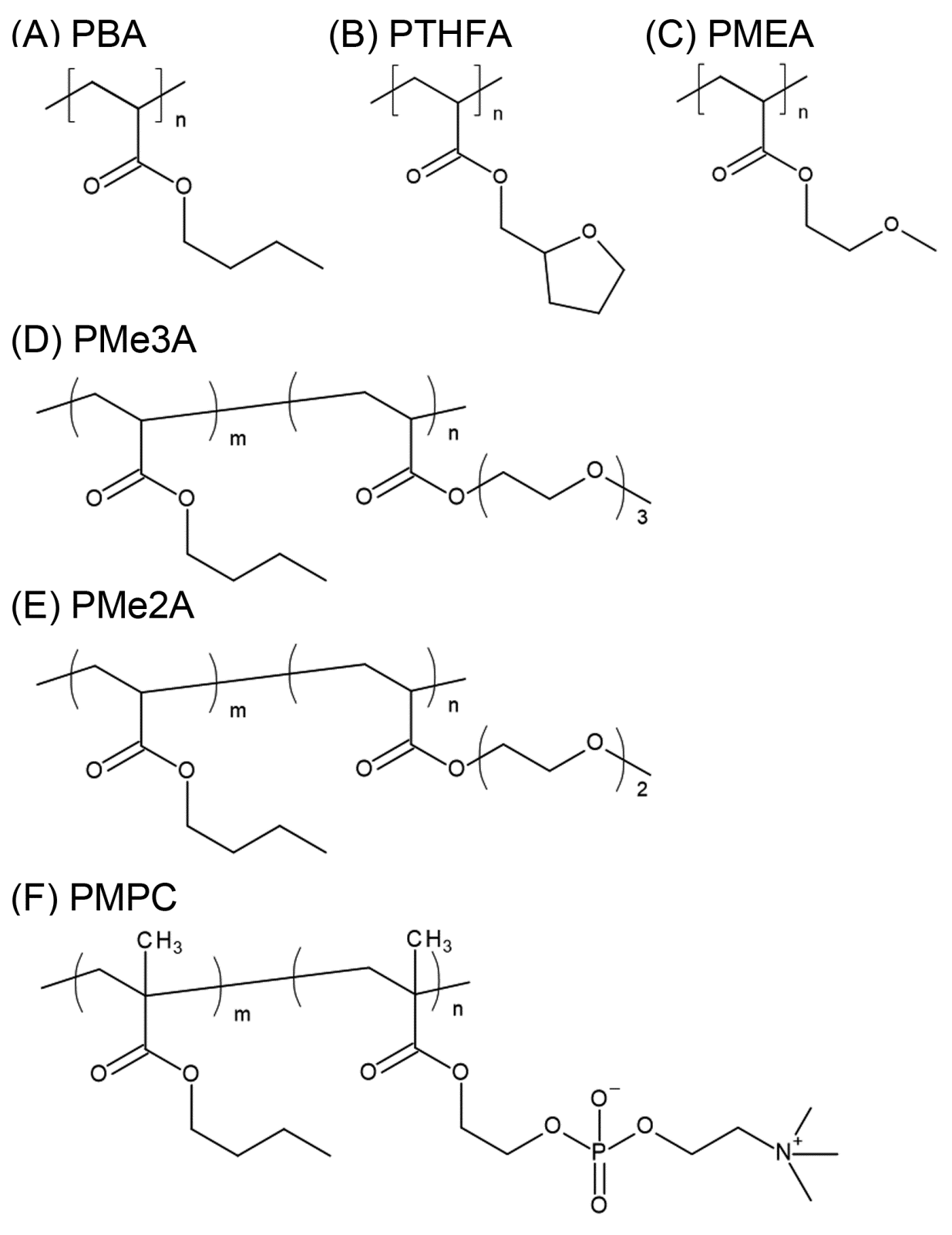

Supplement: S1 File — (Table A) Water content in hydrated PMEA-analogous polymers (wt%). (Figure A) Focal adhesion formation of MDA-MB-231 on PMEA-analogous polymer substrates after 1 day. (Figure B) Focal adhesion formation of HepG2 on the PMEA-analogous polymer substrates after 1 day. (Figure C) Relationship between intermediate water content and protein adsorption. (Figure D) Relationship between intermediate water contents and cell attachment. (Figure E) Chemical structure of PMEA analogous polymers. (DOC) [file pone.0136066.s001.doc]
